# Development and evaluation of an appraisal form to assess clinical effectiveness of adult invasive mechanical ventilation systems

**DOI:** 10.1186/1757-7241-20-45

**Published:** 2012-07-02

**Authors:** Hong Li, Li-Li Chen, Na Li

**Affiliations:** 1 Fujian Provincial Hospital, Fujian Medical University Affiliated Clinical Medical Institute, Nursing School of Fujian Medical University, No 134, East Street, Fuzhou City, Fujian Province, China

**Keywords:** Clinical effectiveness, Appraisal form development, Mechanical ventilation, Nursing, Reliability

## Abstract

**Background:**

Rapid developments in intensive care medicine have made mechanical ventilation an essential method in the resuscitation and comprehensive treatment of critical care patients. This study aimed to develop and evaluate an appraisal form assessing the clinical effectiveness of adult invasive mechanical ventilation systems.

**Methods:**

An appraisal form was designed according to the effectiveness evaluation theory of the American Weapons Systems Effectiveness Industry Advisory Committee (WSEIAC) along with literature review and expert panel review. Content validity of the preliminary form was analyzed in a cohort of 200 patients. Exploratory and confirmatory factor analysis was used to assess appraisal form validity. Discriminate validity of different ventilation outcomes was analyzed by *t* test. Test/retest reliability and inter-scorer reliability were evaluated with 30 patients after a 2-week interval by Cronbach's alpha.

**Results:**

Exploratory factor analysis showed eigenvalues for 3 dimensions (availability, dependability, capability) to be 7.85, 4.43, and 4.22, respectively. Cronbach’s α for internal consistency of the appraisal form was 0.957, and 0.922, 0.961 and 0.937, respectively, for the 3 dimensions. Test-retest reliability of 3 dimensions was 0.976, and 0.862, 0.857, 0.885, respectively. Intra-class correlation coefficient verified test-retest reliability; ICC 0.976 and 0.862, 0.857, 0.885 for 3 dimensions, respectively.

**Conclusions:**

The appraisal form for clinical effectiveness of adult invasive mechanical ventilation systems has high reliability and validity and may be used in clinical setting.

## Introduction

Rapid developments in intensive care medicine have made mechanical ventilation an essential method in the resuscitation and comprehensive treatment of critical care patients [1]. About 80% of patients in intensive care units are reported to require mechanical ventilation, and nursing care of patients receiving mechanical ventilation has become increasingly important, including nurse-led weaning of ventilator patients [2]. While hospitals in other countries may rely on trained respiratory care therapists to assess and care for mechanical ventilation systems [3], in the intensive care units of China, nursing staff especially trained in basic knowledge and techniques of intensive care medicine provide the main care for critically ill patients, including airway care for patients requiring mechanical ventilation. An important role of clinical nursing staff is to correctly assess the effectiveness of mechanical ventilation systems during respiratory intensive care in order to guarantee safe and effective treatment of patients [3-5]. While mechanical ventilation potentially saves patients’ lives, the complexity and diversity of tracheostomy tubes, specific care of surgical sites and other nursing issues such as unplanned intubation and complications may have an impact on its clinical effectiveness [5]. In North American hospitals, while care of the mechanical ventilation system may be in the hands of trained respiratory therapist, airway assessment and management remains a primary responsibility of nurses caring for critically ill adults [5]. Serious concerns of clinical nursing staff and respiratory therapists about mechanical ventilation have led to investigations of the relative effectiveness of ventilation machines, endotracheal tubes and tracheostomy tubes [3-5], as well as the critical care environment [6,7]; personnel issues [8,9]; management of artificial airways [10,11]; and prevention of complications [12-14]. However, current research about nursing evaluation of mechanical ventilation mainly focuses on analyzing and discussing single influential factors, while studies of comprehensive nursing interventions for mechanical ventilation systems are lacking. This may be due in part to the fact that nursing staff may not be responsible for mechanical ventilation systems in some countries, while in China, ICU nurses are solely responsible for mechanical ventilation systems and related patient care. Therefore, the design of an appraisal form to evaluate the comprehensive clinical effectiveness of adult mechanical ventilation systems may provide a valuable assessment tool for critical care nurses responsible for mechanical ventilation administered to patients in intensive care units in China.

Mechanical ventilation systems comprise positive airway pressure ventilation machines connected to patients *via* artificial airways (*i.e.*, endotracheal tubes or tracheotomy) [15]. The Joint Commission (TJC) (formerly the Joint Commission on Accreditation of Healthcare Organizations [JCAHO]), is a US-based hospital accreditation organization that demands regular evaluation of the effectiveness of mechanical ventilation systems along with other equipment used for patient care or diagnostic testing; TJC also requires that regulations and operating procedures must be maintained in written form for professional staff [16]. System effectiveness can be measured by any of several models and one of the most accepted is the effectiveness model advanced by the American Weapons Systems Effectiveness Industry Advisory Committee (WSEIAC) [17]. The WSEIAC model has been used successfully to evaluate the effectiveness of various systems, including radar jamming [18], weapons systems [19], reconnaissance satellites [20], and preventive maintenance of equipment [21]. According to the theory of effectiveness evaluation of the WSEIAC model, there are three dimensions that define system effectiveness [17]. The first dimension of system effectiveness is availability. For mechanical ventilation systems, this means ensuring proper mechanical ventilation by conducting regular system checks, including testing of machine function, setting up proper alarms, maintaining the machine circuit and humidification device and selecting proper endotracheal or tracheotomy tubes [3-5]. Personnel training is also necessary to ensure that all nursing staff members or respiratory therapists possess the essential related knowledge and techniques for machine operation and patient care [21-24]. In addition, the working environment itself can have an impact on nursing quality and the mortality of intensive care patients [1,6,7]. Dependability is the second dimension of system effectiveness in the WSEIAC model, which includes maintaining patency of the airway as an essential nursing intervention [10,11,25,26], securing endotracheal and tracheotomy tubes in a safe position without pressure on the skin [5], reducing intratracheal damage by maintaining the lowest pressure of the endotracheal balloon and preventing air leaks by frequent checking and adjusting as needed and avoiding twisting or pulling on the endotracheal or tracheostomy tubes [27,28]. The airway also must be maintained when patients are being transferred or their position changed [11,29,30]. Patients under mechanical ventilation may also develop physical and mental reactions such as anxiety, fear, pain, sleeping disorders, depression and hallucinations [31,32], requiring that every patient with mechanical ventilation is assessed by nurses to establish an individual nurse-patient communication method and plan [15]. The third dimension of system effectiveness is capability, which is determined by the assessment of complications (*e.g.*, ventilator-associated pneumonia and airway damage), unplanned extubation [33], aspiration [34,35] and patients’ physical and mental reactions [36,37].

Based on the WSEIAC model of system effectiveness, this research aimed to develop and evaluate an effectiveness appraisal form for assessing adult invasive mechanical ventilation systems (EAP-AIMVS), and to apply it for measuring the effectiveness of nursing staff in establishing artificial airways and helping to wean patients from mechanical ventilation. To our knowledge, this is the first system effectiveness appraisal form to be developed for assessing mechanical ventilation systems based on the WSEIAC model.

## Methods

We designed a cross-sectional study to develop and test an appraisal form for evaluating the effectiveness of adult mechanical ventilation systems. This study is divided into two stages: development of the appraisal form and an evaluation stage, including clinical validation of the appraisal form. The Internal Review Board of the Nursing School of Fujian Medical University reviewed the study protocol and approved the study.

### Stage 1: Development stage

#### Items development

The effectiveness appraisal form for mechanical ventilation, including 3 dimensions and 45 items, was formulated based on the WSEIAC model [17], integrated literature review to formulate the item pool, and expert review by a panel of five clinical nurses. Literature review was conducted by the research team; previously published reports regarding scale development and establishing mechanical ventilation system evaluation indexes helped to guide development of the items of the appraisal form [38-46]. The three dimensions of the scale are described as follows: (1) The first dimension is system availability (A), representing the state of the mechanical ventilation system availability at all times (13 items). (2) The second dimension is system dependability (D), representing the correct operation of the mechanical ventilation by nursing staff (19 items). (3) The third dimension is system capability (C), representing the ability of the mechanical ventilation system to achieve goals of respiratory support (13 items). Therefore, system effectiveness (E) is defined as the comprehensive effectiveness of mechanical ventilation system to meet the mission of ventilation within a given time and under intensive care environment.

#### Content validity test

Content validity of the appraisal form was tested by content validity index (CVI), and evaluated by experts as previously described [39]. The panel of experts had 25 members, including 13 experts in clinical intensive care medicine or research and 12 registered nurses, each with more than 10 years of working experience. Four scores were used to evaluate the appraisal form items: 1 = irrelevant, 2 = some relevance, 3 = relevant, 4 = very relevant. Experts assessed every item and the relevance and feasibility of its dimensions, and advanced suggestion and opinions. The appraisal form was modified based on review and suggestions of the experts after deleting six items and integrating six items into other items, a preliminary appraisal form has 33 items in total. CVI of the appraisal form was 0.87 on average, indicating that every item reflects the theme concept of mechanical ventilation system effectiveness evaluation and can be studied further as previously described [40].

#### Preliminary examination

To determine readability and practicability of the appraisal form, 30 patients were assessed by three clinical nurses using the appraisal form. Two items were deleted and three items only partially understood as expressed were revised. As a result, the final EAP-AIMVS had 31 items deemed appropriate for further testing and analysis in the clinical setting.

### Stage 2: Clinical validation of the appraisal form

#### Study subjects

The sample size for multiple factors analysis research should be more than 200 or 10 times the number of items according to a previously established formula [41]. Sample size for this study was established at 200 according to the number of items, progress of this study and feasibility. Patients receiving mechanical ventilation were selected by convenience sampling and 210 ICU patients were enrolled. Inclusion criteria were: patients age ≥18 years with invasive artificial airway (orotracheal or nasotracheal intubation, or tracheotomy.) and assisted mechanical ventilation; conscious patients willing to participate in this study, or unconscious patients with permission of family members. Exclusion criteria were: patients with noninvasive mechanical ventilation, or unwilling to participate in this study; patients without important indicators and patients transferred to other hospitals during ventilation. Ten intensive care units in 8 hospital in Fujian were selected as study locations. Inclusion criteria for ICU nursing staff were: permanent staff with nursing practice qualification certification and having had training for basic concepts, knowledge and techniques of ICU medicine. Nurses who were rotating ICU nursing staff and not permanent staff were excluded. The data collection period was from September 2010 to March 2011. Signed informed consent was received from all participants or their responsible family members.

#### Assessment tool

Demographic data: We recorded gender, age, APACHE II score, type of ventilation machine and artificial airway, time of mechanical ventilation and successful weaning of each patient from mechanical ventilation.

Assessment form scoring: The effectiveness appraisal form for assessing adult invasive mechanical ventilation systems is a nurse-administered assessment tool with three dimensions and 31 items. (Additional file 1: Table S1: Original EAP-AIMVS questionnaire) Four grades were recorded for each item: fully achieved, mostly achieved, hardly achieved and not at all achieved, and scores were 4, 3, 2, and 1, respectively. The higher scores are, the higher the level of clinical effectiveness will be.

### Data collection method

Before collecting data, the effectiveness appraisal form for adult invasive mechanical ventilation system was audited and approved by the hospital internal review committee. Researchers then used the effectiveness appraisal form to assess patients with nonparticipant observation, meaning that except for researchers and coordinators of each study location, nursing staff were not aware of researchers’ identity. Researchers observed every index of the mechanical ventilation system as trainees, collecting data by referring to medical records, and assessed every item of the appraisal form. A total of 210 appraisal forms were filled out, and 210 appraisal forms were returned. Return rate was 100%, and 200 appraisal form were effective, attaining an effectiveness rate of 95.2%.

### Data analysis

All statistical analyses were performed using SPSS 15.0 for Windows statistics software (SPSS Inc, Chicago, IL, USA). Subjects’ demographics and characteristics were summarized as mean with range (min. to max.) for continuous variables and n (%) for categorical variables. Demography statistics used descriptive analysis (frequency, mean, standard deviation and median). Statistical significance was established as P < 0.05.

#### Item analysis

Item analysis assesses relativity between each item and total scores. When the correlation coefficient of each assessment item and total scores was more than 0.9, it was considered redundant. When less than 0.4, the item was deleted as not reflecting the concept to be assessed.

#### Construct validation

Construct validity of the appraisal form was assessed by exploratory factor analysis (EFA) and confirmatory factor analysis (CFA). Exploratory factor analysis evaluates the structure of factors and the consistency between each item and factor structure. Kaiser-Meyer-Olkin Measure of Sampling Adequacy and Bartlett’s Test of Sphericity were used to judge whether factor analysis can be employed. Principle Component Methods was employed to select varimax orthogonal rotation. During factor analysis, guidelines for filtration of assessed items were: (1) each factor’s eigenvalue >1; (2) loading capacity of items with related factor >0.4; (3) each factor at least covers 3 items; (4) tested by scree plot.

To validate matching level of sample data and factor structure of the appraisal form, linear structural equation modeling software [AMOS 17.0, Chicago, SPSS Inc] was employed for confirmatory factor analysis of the appraisal form items that were attained by exploratory factor analysis. Fitting degree was evaluated with *χ*^2^/df, comparative fit index (CFI), normed fit index (NFI), root mean square error of approximation (RMSEA). Model fitting effectiveness is acceptable when *χ*^2^/df < 3, CFI > 0.9, NFI > 0.9, RMSEA < 0.08, according to a previous report [43].

#### Congruent validity

Congruent validity was tested by comparing scores of different groups to see whether there were significant differences, as described in a previous report [44]. This study divided mechanical ventilation patients into a ‘weaned from ventilator successfully’ group and a ‘deceased died during ventilation’ group, and compared the scores of these two groups to determine differences, using discriminate analysis to evaluate the descriptive validity. Results were shown for Wilk’s Lambda test and, and respective sensitivity, specificity.

#### Reliability testing

Internal consistency reliability, test-retest reliability and inter-scorer reliability were selected to evaluate the appraisal form. Cronbach's alpha was calculated to analyze internal consistency of each dimension and the form as a whole. Thirty patients were randomly selected within an interval of 2 weeks to evaluate test-retest reliability by calculating weight coincidence coefficients of these two assessments. Since the appraisal form is a nurse-administered evaluation form, intraclass correlation coefficients were done by 2 scorers evaluating the same 30 patients in the same period to test inter-scorer reliability.

### Ethical considerations

This research was approved by the thesis defense committee for masters degree and by the internal review boards of 8 hospitals in Fujian province whose ICUs were included in the study. Before data collection, conscious patients and family members of unconscious patients were fully informed of the purpose and method of this study, and all provided signed informed consent. All appraisal forms were completed anonymously.

## Results

A total of 200 subjects (142 males/58 females) were enrolled in this study. The mean age was 58.3 years (Range: 16 to 99), with 42 subjects younger than 45 years (yrs), 35 aged between 45 to 55 year, 46 aged between 55 to 65 yrs, 38 aged between 65 to 75 yrs, and 39 older than 75 yrs. The mean time in duration of mechanical ventilation was 216.8 hours (range: 2 to 2880 hours). Ventilation outcomes for the 200 subjects included 123 (61.5%) weaning patients and 77 (38.5%) deceased patients during ventilation. (Table 1)

**Table 1:** Subjects’ demographics and characteristics

**Variables**	**(N = 200)**
Age	58.3 (16 to 99)
< 45 yrs	42 (21.0)
45 – 55 yrs	35 (17.5)
55 – 65 yrs	46 (23.0)
65 – 75 yrs	38 (19.0)
≧ 75 yrs	39 (19.5)
Sex	
Male	142 (71)
Female	58 (29)
APACHE II score	
<10	50 (25.0)
11-20	67 (33.5)
21-30	41 (20.5)
>30	42 (21.0)
Ventilator type	
Servos	42 (21.0)
PB840	60 (30.0)
Vela	36 (18.0)
Drager	62 (31.0)
Artificial airway	
endotracheal tube by mouth	123 (61.5)
endotracheal tube by nose	30 (15.0)
tracheotomy	16 (8.0)
endotracheal tube by mouth + Tracheotomy	31 (15.5)
Duration of mechanical ventilation, hour	216.8 (2 to 2880)
Ventilation outcome	
Weaning	123 (61.5)
Died during ventilation	77 (38.5)

Data were summarized as mean (Range: Min. to Max.) for continuous variables and n (%) for categorical variables.

### Items analysis

The EAP-AIMVS analysis result for 31 items was: 9 items deleted whose relativity with total score was <0.3, and 22 items were reserved for construct validity test.

### Construct validity

The exploratory factor analysis (EFA) used the principle component method for selection and performing varimax rotation for the 200 subjects. EFA results are summarized in Table 2. The KMO index = 0.946 and χ² = 4359 from the Bartlett’s Test of Sphericity (P < 0.01) indicated that this dataset could be applied into EFA. (Results are not shown in tables.) Three factors were selected according to the criteria of Eigenvalue > 1, the three observed eigenvalues greater than 1 were 7.85, 4.43, and 4.22, respectively (with green background). The 22 items were classified into three factors representing the three dimensions of system assessment, as follows: availability (factor 1), dependability (factor 2), and capability (factor 3). Factor loadings were observed from 0.74 to 0.87 in factor 1, from 0.43 to 0.85 in factor 2, and from 0.76 to 0.87 in factor 3. The cumulative variance explained by factors were 75.01%, including 35.68% for factor 1, 20.15% for factor 2, and 19.18% for factor 3 (Table 2).

**Table 2:** **Re-arranged EAP-AIMVS form*via*Exploratory Factor analysis**

**Item**	**Eigenvalue**	**Factor loading**	**Variance explained by factor**
**Factor 1:Availability**	7.85		35.68
1. Check and evaluate function of ventilator		0.87	
2. Select available types and styles of endotracheal or tracheostomy tubes		0.74	
3. Nursing staff has the necessary knowledge and skills to manage machanical ventilation system		0.84	
4. Various policies and procedures related to mechanical ventilation are available		0.85	
5. Environment is suitable for ventilated patient		0.78	
**Factor 2: Dependability**	4.43		20.15
6. Ensure that ventilation tubing is not twisted and that it is adequately supported so as not to pull on ETT/trachi		0.72	
7. Check placement of tube by listening for equal bilateral breath sounds		0.82	
8. Ensure that endotracheal tube or tracheostomy tube is held securely in position but not too tightly to result in pressure area lesions		0.43	
9. Stabilize the tube while turning or moving the patient		0.46	
10. When possible, elevate head of bed to 30°to45° to prevent ventilator-associated pneumonia		0.82	
11. Maintain proper cuff pressure and check if necessary to prevent leakage of air and contaminated secretions		0.81	
12. Suction oropharyngeal and tracheal secretions, more often if necessary to maintain a patent airway		0.83	
13. Oral care at least once a shift and more often if indicated		0.85	
14. Carry out appropriate airway humidification to prevent sticky sputum and keep patient comfortable		0.84	
15. Ventilator circuits are changed weekly or as necessary		0.82	
16. HME filters and end expiratory filters are changed routinely every 24 hours or more frequently if condensation is visible		0.83	
17. Assess patients’ psychological state and help them develop individualized nurse-patient communication method and plan		0.82	
**Factor 3: Capability**	4.22		19.18
18. Tube displacement		0.82	
19. Unplanned extubation		0.76	
20. Airway obstruction		0.85	
21. Ventilator-associated pneumonia		0.82	
22. Adverse psychological reactions		0.87	
Total EAP-AIMVS			75.01

Results of eigenvalues, factor loadings, and variance explained by exploratory factor analysis with varimax rotation.

**Figure 1:**
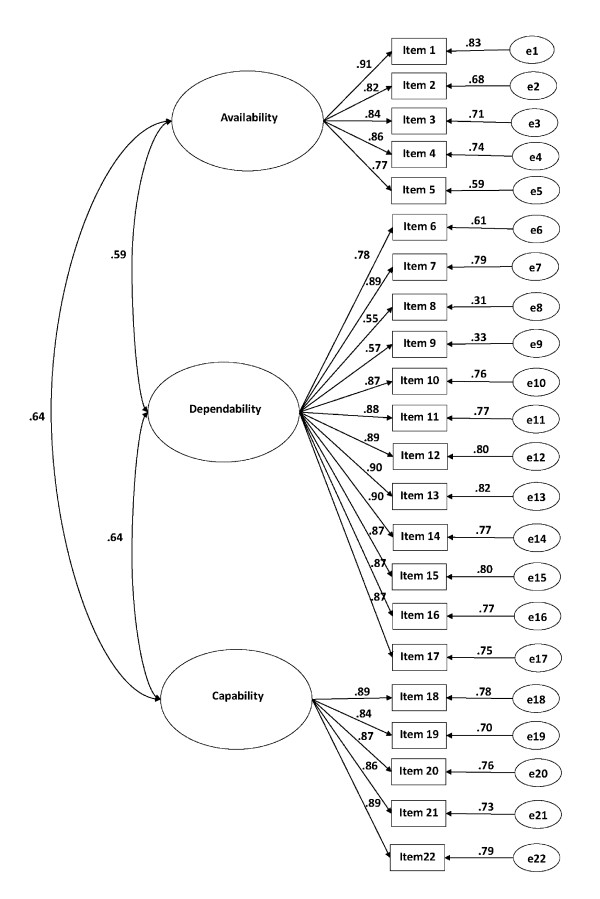
**This figure presents the measurement model evaluated outside the structural equation model (SEM) with correlation coefficients for the relationships among latent variables and standardized betas for the factor loadings.** Availability, dependability, and capability are used as defined from EFA approach. Availability is presented by five indicators (item 1 to 5), dependability is presented by 12 indicators (item 6 to 17), and capability is presented by five indicators (item 18 to 22) The final modified model yielded Chi-square = 34.79 (p < .001), df = 13, *χ*2/df = 2.68, GFI = 0.98, AGFI = 0.94, NFI = 0.95, PNFI = 0.44, PGFI = 0.35, RMSE = 0.07.

Based on the constructed model, the CFA shows the final modified model yielded Chi-square = 34.79 (p < .001), df = 13,*χ*^2^/df = 2.68, GFI = 0.98, AGFI = 0.94, NFI = 0.95, PNFI = 0.44, PGFI = 0.35, RMSE = 0.07 and exhibited good fit indices. (Figure 1)

### Congruent validity

Regarding ventilation outcomes, 123 subjects (61.5%) were weaning and 77 (38.5%) died during mechanical ventilation, and the total EAP-AIMVS score was observed as 80.41 (SD = 6.29) for weaning subjects and 63.13 (SD = 10.19) for those who died during ventilation. (Table 3) The Wilk’s Lambda test from discriminate analysis shows significant discriminate analysis (Wilk’s Lambda = 0.432, P < 0.05), with sensitivity and specificity equal to 91.1% and 77.9%, respectively. (Results are not shown in tables.)

**Table 3:** Summary of distribution of EAP-AIMVS re-arranged after EFA for 200 subjects by ventilation status (N = 200)

**Item**	**Total (N = 200)**	**Weaning (N = 123)**	**Die during ventilation (N = 77)**
**Factor 1:Availability**			
1. Check ventilator function	3.63 ± 0.49	3.84 ± 0.37	3.29 ± 0.45
2. Select available types and styles of endotracheal or tracheostomy tubes	3.6 ± 0.49	3.81 ± 0.39	3.25 ± 0.43
3. Nursing staff has the necessary knowledge and skills to manage mechanical ventilation system	3.66 ± 0.48	3.85 ± 0.36	3.35 ± 0.48
4. Various policies and procedures related to mechanical ventilation are available	3.62 ± 0.49	3.81 ± 0.39	3.31 ± 0.47
5. Environment is suitable for ventilated patient	3.59 ± 0.52	3.78 ± 0.42	3.29 ± 0.53
**Factor 2: Dependability**			
6. Ensure that ventilation tubing is not twisted and that it is adequately supported so as not to pull on the ETT/trachi	3.3 ± 0.69	3.6 ± 0.54	2.82 ± 0.62
7. Check placement of tube by listening for equal bilateral breath sounds	3.32 ± 0.69	3.63 ± 0.52	2.82 ± 0.64
8. Ensure that endotracheal tube or tracheostomy tube is held securely in position but not too tightly to result in pressure area lesions	3.1 ± 0.65	3.34 ± 0.54	2.7 ± 0.61
9. Stabilize the tube while turning or moving the patient	3.12 ± 0.71	3.39 ± 0.6	2.68 ± 0.66
10. When possible, elevate head of bed to 30° to 45° prevent ventilator-associated pneumonia	3.29 ± 0.69	3.58 ± 0.57	2.82 ± 0.6
11. Maintain proper cuff pressure and check if necessary to prevent leakage of air and contaminated secretions	3.26 ± 0.72	3.58 ± 0.57	2.74 ± 0.62
12. Suction oropharyngeal and tracheal secretions, more often if necessary, to maintain a patent airway	3.34 ± 0.68	3.66 ± 0.51	2.83 ± 0.62
13. Oral care at least once a shift and more often if indicated	3.3 ± 0.66	3.61 ± 0.51	2.81 ± 0.59
14. Carry out appropriate airway humidification to prevent sticky sputum and keep patient comfortable	3.28 ± 0.69	3.58 ± 0.54	2.79 ± 0.61
15. Ventilator circuits are changed weekly or as necessary	3.3 ± 0.71	3.61 ± 0.55	2.79 ± 0.64
16. HME filters and end expiratory filters are changed routinely every 24 hours or more frequently if condensation is visible	3.29 ± 0.69	3.59 ± 0.54	2.81 ± 0.63
17. Assess patients’ psychological state and help them develop individualized nurse-patient communication method and plan	3.28 ± 0.7	3.58 ± 0.57	2.81 ± 0.63
**Factor 3: Capability**			
18. Tube displacement	3.16 ± 1.1	3.69 ± 0.68	2.31 ± 1.1
19. Unplanned extubation	3.4 ± 0.9	3.8 ± 0.55	2.74 ± 0.95
20. Airway obstruction	3.36 ± 0.93	3.75 ± 0.58	2.73 ± 1.03
21. Ventilator-associated pneumonia	3.2 ± 1.03	3.63 ± 0.69	2.51 ± 1.11
22. Adverse psychological reactions	3.11 ± 1.08	3.58 ± 0.71	2.35 ± 1.13
Total EAP-AIMVS	73.76 ± 11.62	80.41 ± 6.29	63.13 ± 10.19

EFA, Exploratory Factor analysis.

Data were summarized as mean ± Standard deviations.

### Reliability

Internal consistency reliability of the appraisal form by Cronbach’s α was 0.957 and that for availability, dependability and capability were 0.922, 0.961 and 0.937, respectively. Test-retest reliability of the appraisal form by Kappa is 0.976, and for the three dimensions were 0.862, 0.857, 0.885, respectively. Inter-scorer reliability of the appraisal form, intra-class correlation coefficient verified test-retest reliability; ICC of the appraisal form was 0.976, and for the three dimensions, respectively, 0.862, 0.857, 0.885.

## Discussion

In this 15-month study, we designed, developed and tested an appraisal form for assessing the effectiveness of adult invasive mechanical ventilation systems, the EAP-AIMVS. Our design was based on the WSEIAC model of system effectiveness [17], which had been successfully applied to measure effectiveness of other types of systems [18-21]. To our knowledge, this is the first appraisal form developed for mechanical ventilation systems using the WSEIAC model. Testing of the appraisal form with a convenience sample of 200 critical care patients on mechanical ventilation revealed that the three dimensions of effectiveness according to the WSEIAC model, namely system availability, dependability, and capability, had acceptable internal consistency (overall 0.957, and for the three dimensions, 0.922, 0.961 and 0.937, respectively), reliability, and test-retest reliability. As such, this indicates that the appraisal form can feasibly be applied to the assessment of mechanical ventilation systems by the responsible staff in clinical settings.

Briefly, we must note possible differences in the intensive care clinical setting in China compared to those in other countries. To ensure providing optimum therapeutic requirements for critically ill patients, patients are placed together in a dedicated area in single units of about 15-square meters with necessary equipment (*e.g.*, bedside and central monitors, multi-function breathing and electrocardiograph machines, defibrillators, pacemakers, infusion pumps and equipment for tracheal intubation and tracheotomy). Nurses specially trained in basic knowledge and techniques of intensive care medicine are the primary care staff for critically ill patients. Medical and nursing staff members working independently are responsible for allocation of all equipment and nurses are directly responsible for airway care for patients requiring mechanical ventilation, including the system, all related interventions and weaning from the ventilator system.

### The appraisal form has a solid theoretical basis

The design and development of a research tool requires a powerful theoretical basis [45]. The appraisal form for effectiveness of mechanical ventilation systems as advanced in this research, and which relied on the WSEIAC model as a basic framework complemented by integrated literature review and expert evaluation, combined the three dimensions of availability, dependability and capability to evaluate essential connections and related factors of mechanical ventilation systems, including pre-use state, functioning procedure and results, reflecting the quality control of nursing from pre-use until end-stage. The appraisal form is intended to assist clinical nurses in recognizing key indicators of mechanical ventilation effectiveness. Effectiveness of nursing intervention for the whole procedure or different stages can be assessed by using either the whole appraisal form or its subscales, as suggested by Noar (2003) [47].

### The appraisal form has good construct validity

As evaluated in the second part of this study, the appraisal form exhibited good construct validity. The three common factors, corresponding to the three dimensions of system effectiveness in the WSEIAC model, were extracted *via* exploratory factor analysis, and its accumulated variance contribution rate was 75.01%. Factor classification was followed with the varimax orthogonal rotation of each item, which was even and clear, and the loading capacity of items with related factors was more than 0.4. This indicates good construct validity of the newly designed appraisal form. Limitations of estimating relationships between exploratory factor analysis and variables can indicate uncertainty about the relationships. Therefore, after using exploratory factor analysis as a base, this research then used confirmatory factor analysis to test the level of fitting of the three dimensions model structure with the structural equation model as a foundation. Results of confirmatory factor analysis proved that the three dimensions structure of the appraisal form is highly consistent with the WSEIAC model theoretical framework. This result also reinforced the construct validity of the appraisal form.

### The appraisal form has good congruent validity

Congruent validity demonstrates how a scale differentiates characteristics of different groups of subjects [44]. Esteban and Anzueto (2002) conducted an extensive, foresighted and multi-center research study, finding that patients’ survival rate and prognosis were influenced by different factors of pre-use of mechanical ventilation, and complications and management of patients in mechanical ventilation [47]. As an example of classical test theory, results of that study demonstrated that the assessment index, including management of mechanical ventilation, nursing management of airways and development of complications, influences the prognosis of patients [47]. Therefore, it is commonly understood that the poorer the prognosis of ventilation in a given patient, the lower the effectiveness score would be. Congruent validity demonstrates whether or not the appraisal form reflects differences between factors. In the present study, results showed that patients weaned from the ventilator successfully had higher effectiveness scores than the patients who died during ventilation (t = 13.38,P < 0.05), indicating acceptable congruent validity of the appraisal form.

### The appraisal form demonstrated good reliability

Reliability refers to the level of credibility and stability of a scale and its evaluation results. Reliability can be evaluated by various means. This study reviewed internal consistency reliability, test-retest reliability and inter-scorer reliability. Cronbach’s α is an estimate of internal consistency and will typically increase as relationships between the test items increase. In the present study, Cronbach’s α of the whole appraisal form and its different dimensions achieved the measurement model of more than 0.7, which indicates internal consistency reliability of the appraisal form, in keeping with previously published results [48]. Our result indicates good reliability of the effectiveness appraisal form and the homology of items that form each dimension. Test-retest reliability and inter-scorer reliability also achieved the measurement model of more than 0.75 [49]. It is shown that the variance of these two tests of the appraisal form is relatively low and stable, which indicates that the appraisal form is suitable for use by different clinical assessors.

Prior to actually designing the EAP-AIMVS, we theorized that availability of a standardized form for assessing the effectiveness of adult invasive mechanical ventilation would be a valuable adjunct for nurses in Chinese hospitals caring for ventilated critical care patients, not only to help address nursing issues but to provide safer care for patients. This was the rationale for developing the EAP-AIMVS as a practical, theory-based form for appraising effectiveness of mechanical ventilation systems, and during development we considered exactly how an effectiveness appraisal form might help to ensure patient safety and reduce complications, morbidity and mortality. Other nursing interventions reported in the literature provided encouragement. In a study documenting the need for education of nurses and other medical professionals who care for ventilated patients, the need for additional education and training regarding clinical practice in intensive care was expressed by 62% of medical staff, including intensivists and 42% of nurses [1]. Although having an effectiveness appraisal form will not take the place of education and training, the effectiveness evaluation can serve as a readily available guide or checklist that can be followed by ICU caregivers to help ensure safe, effective ventilation and efficient monitoring. Studies of weaning from mechanical ventilation illustrate another aspect of standardizing care. Because prolonged mechanical ventilation is associated with high morbidity, especially ventilator associated pneumonia and lung injury, and increased mortality, patients receiving mechanical ventilation may actually spend 40% of the time being weaned from ventilation; prolonged ventilation may also preclude patients’ recovery from their primary critical illness [50]. The investigators in that study recommended surveying practitioners and multidisciplinary teams to collect the best ways to put optimal weaning methods into practice and then apply them [50]. Relative to weaning methods, routine application of a nurses’ protocol-directed weaning procedure applied in a French hospital resulted in improved outcomes and clinical benefits in patients requiring more than 48 hours of mechanical ventilation [2]. In our study, routinely following a simple, step-by-step appraisal form for mechanical ventilation effectiveness allowed more than 60% of patients to be weaned successfully, helped to prevent complications and reassuring staff that proper procedures had been carried out for system effectiveness, which implies patient safety. We made sure that effectiveness criteria included items such as ventilator circuits and secretion management, which have been shown to influence rates of ventilator-associated pneumonia [13]. We also included the intensive care physical environment as a factor in the appraisal (Item 5, Table 3) since a healthy environment is needed to protect against airborne infection and prevent cross-infection; hand washing and aseptic technique must be reinforced, air quality must be monitored, visitors restricted and ventilation equipment needs disinfecting on a regular basis. Also, a 28-day international study of ventilated patient characteristics and outcomes suggested that survival was associated not only with baseline factors present at the inception of mechanical ventilation but the development of complications and the management of patients [47]. Thus, future research on mechanical ventilation effectiveness must evaluate patient data, including complications, unexpected developments and outcomes, at baseline and during the course of mechanical ventilation.

### Implications for practice

Results of the present study have provided an evaluation form by which nursing staff or other responsible staff such as respiratory therapists can evaluate the effectiveness of mechanical ventilation systems and related care aspects of nursing interventions for mechanical ventilation patients. Specifically, the developed form can help clinical nursing staff to differentiate key indexes and increase work efficiency. Future research should focus on progressive evaluation of mechanical ventilation efficiency in China and abroad, and collect clinical practice data for ventilation technique and management of artificial airways. Guiding principles for evaluation of mechnical ventilation systems can be improved and progressive development of appraisal forms may ultimately include software applications that evaluate each item according to individual patients’ clinical situation.

### Limitations

First, the appraisal form developed in this study for assessing the effectiveness of mechanical ventilation was not developed on the basis of an existing scale but was an initial attempt to develop an original appraisal form and evaluate its suitability as an assessment tool. The sample was also relatively small. Assessment items of the appraisal form therefore require further evaluation with a larger clinical sample. Secondly, convenience sampling method was used and its representativeness is limited. Sampling range and quantity should be broadened in future research, and the appraisal form will be further developed and improved through clinical evidence and progressive mechanical ventilation research.

## Conclusions

The theory-based appraisal instrument to assess nursing interventions for mechanical ventilation systems demonstrates acceptable internal consistency, inter-scorer reliability, and test-retest reliability and can reasonably guide clinical nursing staff to evaluate essential monitoring techniques and factors related to mechanical ventilation. The appraisal form is appropriate for use in the clinical setting and its application may help to improve the effectiveness and performance of adult mechanical ventilation systems as well as helping to provide safe, effective treatment for patients under intensive care.

## Competing interests

The authors declare there is no competing interests.

## Authors’ contributions

LH was responsible for conception, overall design, technical support and content revision. CL was responsible for project implementation, data collection and data analysis. LN was responsible for data organization and analysis. All authors read and approved the final manuscript.

## Supplementary Material

Additional file 1 **Table S1.** Original EAP-AIMVS questionnaire. Click here for file
