# Supplementary material for: Development and evaluation of an appraisal form to assess clinical effectiveness of adult invasive mechanical ventilation systems
Source: Scand J Trauma Resusc Emerg Med. 2012 Jul 2;20:45. doi: 10.1186/1757-7241-20-45 (PMC3419130; doi:10.1186/1757-7241-20-45)
Supplement: Additional file 1 — Table S1. Original EAP-AIMVS questionnaire. [file 1757-7241-20-45-S1.doc]

Supplementary Table 1: Original EAP-AIMVS questionnaire.

| **Item** | Fully achieved  **（4）** | Mostly achieved  **（3）** | Hardly achieved  **（2）** | Not at all achieved  **（1）** |
| --- | --- | --- | --- | --- |
| **Dimension 1:Availability** | | | | |
| 1. Check ventilator function （r=0.602） | □ | □ | □ | □ |
| 2. Select available types and styles of endotracheal or tracheostomy tubes （r=0.658） | □ | □ | □ | □ |
| 3. Ventilator proximal leak check（r=0.264） | □ | □ | □ | □ |
| 4. Nurses Specially trained（r=0.269） | □ | □ | □ | □ |
| 5. Nursing staff has the necessary knowledge and skills to manage mechanical ventilation system（r=0.590） | □ | □ | □ | □ |
| 6. Various policies and procedures related to mechanical ventilation are available（r=0.587） | □ | □ | □ | □ |
| 7. Environment is suitable for ventilated patient（r=0.568） | □ | □ | □ | □ |
| 8. Standard handwashing（r=0.121） | □ | □ | □ | □ |
| 9. Restrictions to access personnel（r=0.264） | □ | □ | □ | □ |
| **Dimension 2: Dependability** | | | | |
| 10. Ensure that ventilation tubing is not kinked and is adequately supported so as not to drag on ETT/trachi（r=0.751） | □ | □ | □ | □ |
| 11. Check placement of tube by listening for equal bilateral breath sounds（r=0.818） | □ | □ | □ | □ |
| 12. Ensure that endotracheal tube or tracheostomy tube is held securely in position but not too tightly to result in pressure area lesions（r=0.624） | □ | □ | □ | □ |
| 13. Take care of the tube while turning or moving the patient（r=0.625） | □ | □ | □ | □ |
| 14. When possible, elevate head of bed to 30°to 45° to prevent ventilator-associated pneumonia（r=0.794） | □ | □ | □ | □ |
| 15. Maintain proper cuff pressure and check if necessary to prevent leakage of air and contaminated secretions（r=0.819） | □ | □ | □ | □ |
| 16. Suction oropharyngeal and tracheal secretions, more often if necessary to maintain a patent airway（r=0.817） | □ | □ | □ | □ |
| 17. Oral care at least once a shift and more often if indicated（r=0.820） | □ | □ | □ | □ |
| 18. Timely monitor and record the pipeline（r=0.262） | □ | □ | □ | □ |
| 19. Carry out appropriate airway humidification to prevent sticky sputum and keep patient comfortable（r=0.818） | □ | □ | □ | □ |
| 20. Ventilator circuits are changed weekly or if necessary（r=0.795） | □ | □ | □ | □ |
| 21. HME filters and end expiratory filters are changed routinely every 24 hours or more frequently if there is condensation visible（r=0.779） | □ | □ | □ | □ |
| 22. Assess patient’s psychological state and help to develop individualized nurse-patient communication method and plan（r=0.781） | □ | □ | □ | □ |
| **Dimension 3: Capability** | | | | |
| 23. Tube displacement（r=0.796） | □ | □ | □ | □ |
| 24. Human-machine coordination（r=0.264） | □ | □ | □ | □ |
| 25. Unplanned extubation（r=0.767） | □ | □ | □ | □ |
| 26. Airway obstruction（r=0.730） | □ | □ | □ | □ |
| 27. Airway injury（r=0.111） | □ | □ | □ | □ |
| 28. Ventilator-dependent（r=0.120） | □ | □ | □ | □ |
| 29. Ventilator-associated lung injury（r=0.108） | □ | □ | □ | □ |
| 30. Ventilator-associated pneumonia（r=0.735） | □ | □ | □ | □ |
| 31. Adverse psychological reactions（r=0.728） | □ | □ | □ | □ |
| Total EAP-AIMVS |  | | | |
